# Antihypertensive effects of the combined extract of *Sorghum bicolor*, *Vigna angularis*, and *Eleusine coracana* in spontaneously hypertensive rats

**DOI:** 10.1038/s41598-024-51364-5

**Published:** 2024-01-08

**Authors:** Eunwoo Jeong, Damin Yun, Youjin Baek, Hyun-Joo Kim, Hyeon Gyu Lee

**Affiliations:** 1 Department of Food and Nutrition, Hanyang University, 222 Wangsimni-ro, Seongdong-gu, Seoul, 04763 Korea; 2 Department of Central Area Crop Science, National Institute of Crop Science, Wanju-Gun, 55365 Korea

**Keywords:** Plant sciences, Diseases, Endocrinology

## Abstract

This study investigated the antihypertensive effects of the combined extract of sorghum, adzuki bean, and finger millet (SAFE) on spontaneously hypertensive rats. The rats were divided into four groups (*n* = 8): WKY, SHR, SAFE (500 mg/kg SAFE), and CAP (50 mg/kg captopril). SAFE significantly decreased the lean-to-fat mass ratio with no notable changes in body weight, food intake, or food efficiency ratio, and it effectively lowered both systolic and diastolic blood pressures, comparable to CAP. Moreover, it significantly reduced the cardiac mass index and alleviated cardiac fibrosis. SAFE did not induce hepatotoxicity, as indicated by the maintenance of aspartate aminotransferase and alanine aminotransferase levels in the normal range, confirming its safety. Taken together, these findings suggested that SAFE can be used as a dietary supplement for blood pressure regulation and cardiovascular disease prevention.

## Introduction

Hypertension, often called a “silent killer,” is a leading cause of mortality and a major health concern worldwide. Moreover, 1.28 billion adults aged 30–79 are estimated to have high blood pressure, indicating that three in ten adults suffer from hypertension worldwide. Approximately two–thirds of these individuals reside in low- and middle-income countries^1^. Hypertension is the most significant risk factor for cardiovascular diseases. Although no obvious symptoms are observed in the early stages, if not properly treated and managed, hypertension can lead to stroke, kidney disease, heart failure, and death from cardiovascular disease, posing a substantial socioeconomic burden on the affected patients^2^. Therefore, prevention and treatment of this condition are important to maintain public health.

Cardiac hypertrophy and fibrosis are common consequences of hypertension. Prolonged high blood pressure increases the heart workload, leading to cardiac remodeling via structural and functional changes, including cardiac hypertrophy and myocardial fibrosis, in which the heart muscles become large and thick^3^. Cardiac hypertrophy and fibrosis are early indicators of heart disease progression that ultimately lead to heart failure, myocardial infarction, and other cardiovascular diseases. Therefore, cardiac hypertrophy and fibrosis alleviation are necessary to treat hypertensive complications.

Essential hypertension, which accounts for approximately 90% of people with high blood pressure, occurs without a clear cause, and it is treated using medications along with dietary and lifestyle modifications. Antihypertensive drugs, such as thiazide diuretics, calcium channel blockers, and angiotensin-converting enzyme inhibitors, exhibit therapeutic benefits for clinical use^4^. However, these drugs exhibit side effects such as angioedema and cough^5^. These side effects may reduce the patient's adherence to prescribed doses and increase the severity of hypertension and the cost of treatment. Consequently, interest in exploring natural bioactive substances as alternative approaches for managing hypertension has increased.

Grains and legumes, prominently used in traditional medicine, are functional food ingredients due to their bioactive properties^6^. Sorghum (*Sorghum bicolor*), adzuki bean (*Vigna angularis*), and finger millet (*Eleusine coracana*) have high amounts of phenolic compounds and distinct profiles compared with other cereals. Sorghum is a rich source of 3-deoxycyanidins and tannins, which are not commonly found in other cereals^7,8^. Previous studies have reported the antioxidant, antihyperlipidemic, and antihypertensive effects of sorghum extracts. Adzuki beans contain catechin, protocatechin, gallic acid, chlorogenic acid, rutin, and quercetin glycosides. Previous studies have highlighted the antihypertensive activity of adzuki bean extract, including its effects on macrophage infiltration, vascular oxidative stress, inflammation, blood pressure elevation, and nitric oxide production^9,10^. Finger millet has high levels of specific amino acids (valine, lysine, and methionine), calcium, and polyphenols^11^. We previously demonstrated the blood pressure-lowering effects of finger millet and adzuki bean extracts through their antioxidant activity and modulation of the renin–angiotensin system^12,13^.

Considering the efficacy of the individual extracts, we hypothesized that the combined extract of sorghum, adzuki beans, and finger millet may exert antihypertensive effects. However, to our knowledge, the antihypertensive effects of the combination of grain and legume extracts have not yet been elucidated. Furthermore, no studies have explored the effects of these extracts on cardiac hypertrophy and fibrosis. Therefore, in this study, we aimed to investigate the blood pressure-lowering and cardiac structure-protective effects of a combined ethanol extract of sorghum, adzuki bean, and finger millet (SAFE) in spontaneously hypertensive rats.

## Materials and methods

### Sample preparation

Sorghum (*S. bicolor* Sodamchal), adzuki bean (*V. angularis* Arari), and finger millet (*E. coracana* Finger 1-ho) were obtained from the National Institute of Crop Science (Rural Development Administration, Suwon, Korea). SAFE was prepared as follows: Sorghum, adzuki beans, and finger millet were ground into a powder and mixed at 35:35:30 (w/w/w). The mixing ratio was selected based on preliminary experiments on the inhibitory effects of ACEs. The mixture was extracted with 99% ethanol and stirred overnight at room temperature. Then, the extract was filtered using Whatman No. 2 filter paper, evaporated using a rotary vacuum evaporator (Eyela, Tokyo, Japan) at 50 °C, and kept at − 80 °C until further use.

### Animals

Six-week-old male Wistar-Kyoto rats (WKY) and spontaneously hypertensive rats (SHR) were provided by Central Lab Animal, Inc. (Seoul, Korea). All rats were housed at a fixed temperature (22 ± 1 °C) and relative humidity (50 ± 10%) with a 12 h light cycle. A standard diet (PicoLab Rodent Diet 20; LabDiet, St. Louis, MO, USA) and water were provided ad libitum. After adaptation for one week, the rats were divided into four groups (*n* = 8): (a) WKY, (b) SHR, (c) SAFE (500 mg/kg body weight SAFE), and (d) captopril (CAP; 50 mg/kg body weight CAP). SAFE was dissolved in 10% Kolliphor (BASF, Ludwigshafen, Germany). The rats were orally administered saline, SAFE, or CAP for six weeks. Animal experiments were approved by the Institutional Animal Care and Use Committee of Hanyang University (approval number HY-IACUC-21-0047). In addition, all procedures for animal experiments described in this study were performed in accordance with the HY-IACUC guidelines for the care and use of laboratory animals and ARRIVE guidelines.

### Growth performance and body composition

Body weight and food consumption were measured once and twice weekly, respectively. The food efficiency ratio was calculated as the ratio of weight gain to total food intake. After six weeks, the rats were fasted for 12 h and anesthetized via an intraperitoneal injection of xylazine (15 mg/kg body weight), followed by ketamine (100 mg/kg body weight). Body composition, including fat and lean mass and bone mineral density, was assessed using dual-energy X-ray absorptiometry (InAlyzer Medikors, Seongnam, Korea). The fat-to-lean mass ratio was calculated as fat mass (g)/lean mass (g) × 100.

### Blood pressure measurement

During the experimental period, systolic blood pressure (SBP) and diastolic blood pressure (DBP) were measured weekly using the tail-cuff method with a noninvasive blood pressure monitor. To minimize the error range of blood pressure measurements, the rats were placed in a BP-2000 holder (Visitech Systems, Apex, NC, USA) and stabilized in a heating chamber at 36 °C for 5 min. The blood pressure of each rat was measured at least three times.

### Blood biochemical analysis

Blood samples were collected via cardiac puncture under anesthesia. Aspartate aminotransferase (AST) and alanine transaminase (ALT) levels were assessed using commercial kits (Asan Pharmaceutical, Seoul, Korea) according to the manufacturer’s instructions.

### Histological analysis of the heart

Isolated hearts were weighed, fixed with 10% formalin (Sigma-Aldrich, St. Louis, MO, USA), dehydrated, and paraffin-embedded to prepare the tissue sections. Each tissue sample was stained with hematoxylin and eosin before dehydration and mounting. The stained areas were observed under a microscope at 100× magnification.

### Statistical analyses

Data are expressed as the mean ± standard error of the mean. Statistical analyses were performed using the GraphPad Prism 9 software (GraphPad Software, La Jolla, CA, USA). The data were analyzed using a one-way analysis of variance, followed by Fisher’s least significant difference test. Statistical significance was set at *p* < 0.05.

### Ethical approval

Animal experiments were approved by the Institutional Animal Care and Use Committee of Hanyang University (approval number HY-IACUC-21-0047). In addition, all procedures for animal experiments described in this study were performed in accordance with the HY-IACUC guidelines for the care and use of laboratory animals and ARRIVE guidelines.

## Results and discussion

### Body weight, food intake, and food efficiency ratio

**Figure 1:**
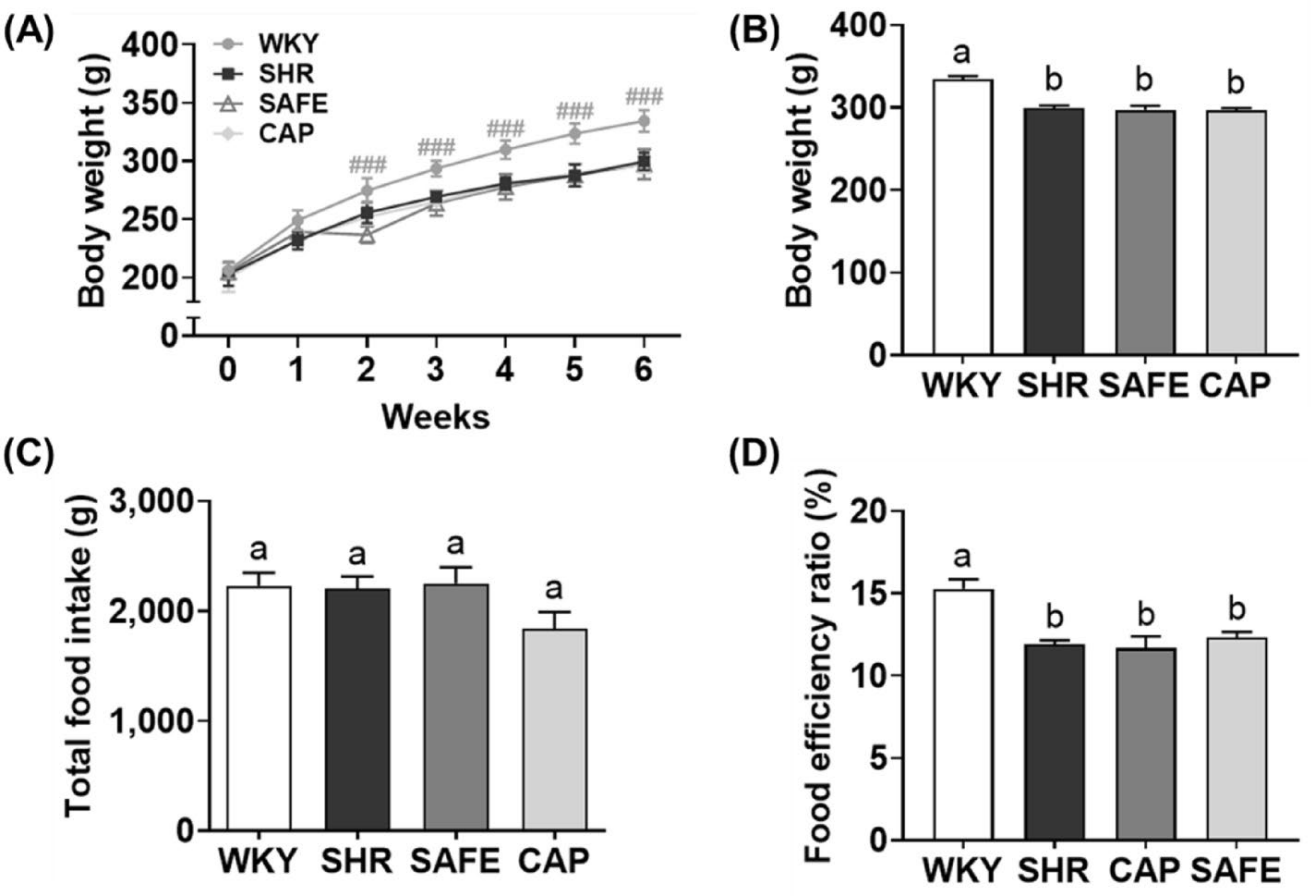
(**A**) Weekly body weight, (**B**) final body weight, (**C**) total food intake, and (**D**) food efficiency ratio of rats supplemented with a combined extract of sorghum, adzuki bean, and finger millet (SAFE). Data are expressed as the mean ± standard error of the mean (SEM; *n* = 8). Statistical analyses were performed using one-way analysis of variance (ANOVA) with the Fisher’s least significant difference (LSD) test. Values with different letters indicate significant differences at *p* < 0.05.

As shown in Fig. 1, hypertensive rats (SHR, SAFE, and CAP) had a lower body weight than that of WKY rats (*p* < 0.05). No difference in food intake was observed among the groups, and the food efficiency ratios of the hypertensive groups were lower than those of the WKY rats because the hypertensive groups gained less weight than the WKY rats. This phenomenon is consistent with the results of previous studies on hypertensive rat strains^14^. Following six weeks of SAFE treatment, no differences were observed in the body weight and food efficiency ratio between the SAFE and SHR groups, even in the CAP group. These results are consistent with previous reports that supplementation with adzuki bean and finger millet extracts did not change the body weight or feed efficiency ratio compared to the hypertensive control groups^12,13^. Therefore, SAFE did not affect the body weight or feed intake in hypertensive rats.

### Body composition

**Figure 2:**
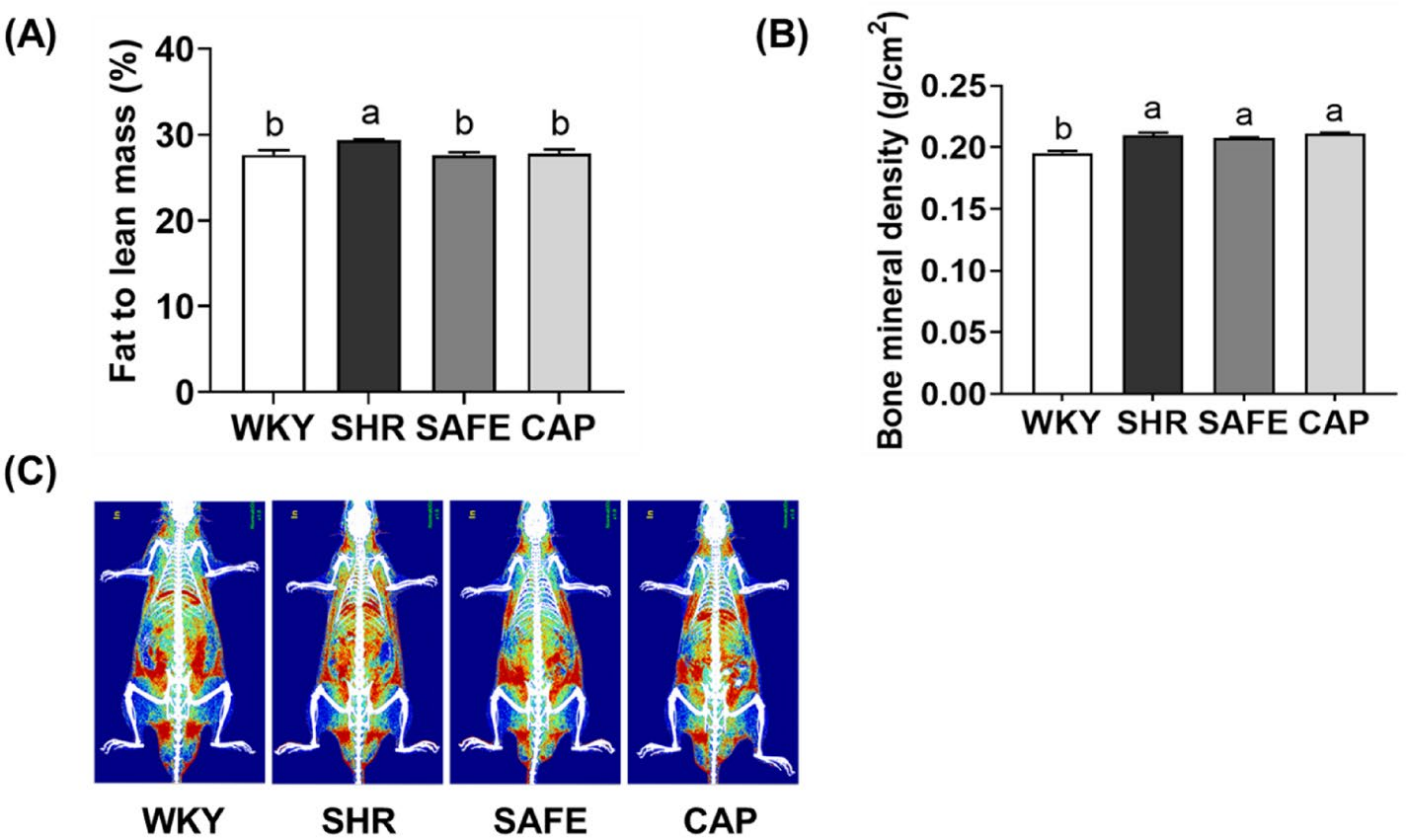
(**A**) Ratio of fat-to-lean mass (%), (**B**) bone mineral density, and (**C**) radiograph of rats supplemented with SAFE. Data are expressed as the mean ± SEM (*n* = 8). Statistical analyses were performed using a one-way ANOVA with the Fisher’s LSD test. Values with different letters indicate significant differences at *p* < 0.05.

After six weeks of SAFE treatment, fat and lean mass and bone mineral density were measured using DEXA. As illustrated in Fig. 2, SHR exhibited a significantly higher fat-to-lean mass ratio than that of WKY rats (*p* < 0.05). SAFE and CAP significantly decreased the fat-to-lean mass ratio compared with that of SHR (*p* < 0.05). In addition, the bone mineral density of the hypertensive groups (SHR, SAFE, and CAP) was significantly higher than that of the WKY group (*p* < 0.05). However, SAFE and CAP did not significantly alter the bone mineral density compared to that of SHR (*p* > 0.05).

The individual constituents of SAFE inhibited fat accumulation and promoted lean mass both in vitro and in vivo^15–17^. However, this study demonstrated a significant reduction in the fat-to-lean mass ratio in a rat model of hypertension. These findings provide primary data on the effects of multigrain extracts on hypertension models.

Hypertension can reduce bone mineral density due to changes in mineral metabolism^18^. Sorghum is abundant in potassium and phosphorus; adzuki beans are rich in magnesium, folate, and potassium; and finger millet is the only grain with a calcium content more than three times that of milk^19–21^. Given these properties, we evaluated the effects of SAFE, a mineral-rich multigrain extract, on bone mineral density; however, no significant differences were observed. Contrary to our expectations, higher bone mineral density was observed in SHR rats than in WKY rats. This inconsistency might be attributed to variations in SHR subtypes, as low bone mineral density is typically associated with aging or stroke-prone spontaneously hypertensive rats^22,23^. In addition, the use of ethanol as an extraction solvent may have resulted in minimal mineral extraction. Hence, future investigations on the effects of SAFE on bone metabolism should involve appropriate animal models and extraction solvents.

### Blood pressure

**Figure 3:**
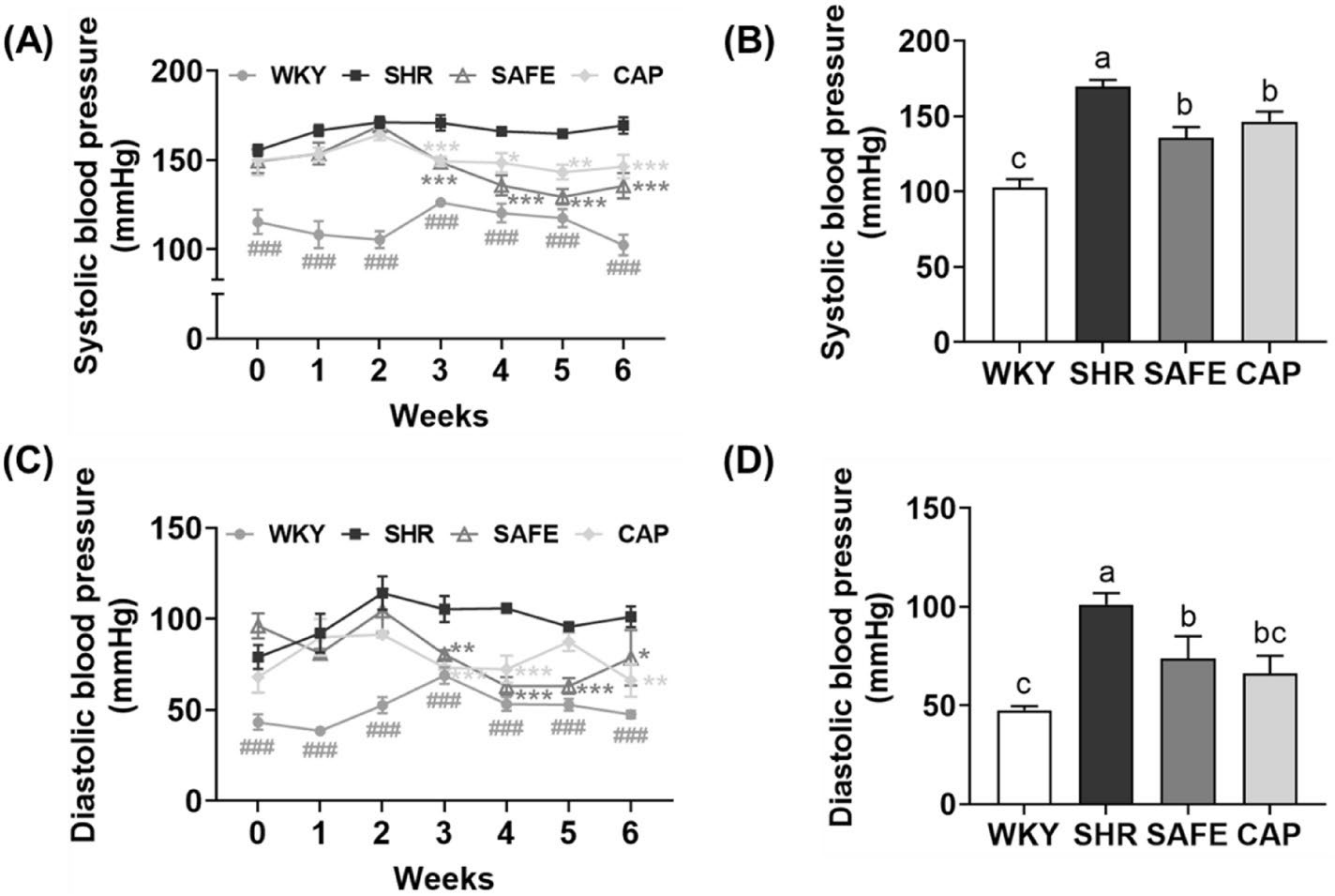
(**A**) Weekly systolic blood pressure, (**B**) systolic blood pressure at week 6, (**C**) weekly diastolic blood pressure, and (**D**) diastolic blood pressure at week 6 of rats supplemented with SAFE. Data are expressed as the mean ± SEM (*n* = 8). Statistical analyses were performed using a one-way ANOVA with the Fisher’s LSD test. Values with different letters indicate significant differences at *p* < 0.05.

To evaluate the blood pressure-lowering effect of SAFE, we measured the SBP and DBP of rats using the tail-cuff method. As depicted in Fig. 3, the initial SBP of all hypertensive groups (SHR, CAP, and SAFE) was in the range of 149.5–155.6 mmHg, which was significantly higher than that of WKY (115.5 mmHg) (*p* < 0.05). This observation confirmed the successful establishment of the hypertension model. Throughout the experiment, WKY maintained the lowest SBPs, whereas SHR exhibited the highest SBP. Oral administration of SAFE resulted in significant SBP reductions beginning at week 3 compared to those in SHR. In the final week of the experiment, the SAFE group had a significantly lower SBP, by 20% (*p* < 0.05), than in the SHR group. Similar findings were observed in the CAP group with no significant difference between SAFE and CAP. The DBP results were consistent with the SBP results, which showed a blood-lowering effect of SAFE over 6 weeks.

We have previously reported a blood pressure-lowering effect of adzuki bean and finger millet extracts compared to that in the control group due to their ability to inhibit ACE and regulate the renin–angiotensin system^12,13^. In addition, sorghum extract inhibits angiotensin-converting enzymes and has a high antioxidant capacity due to its high phenolic compound content^24^. Although the exact mechanism of the hypotensive effect of SAFE is unclear, the ACE inhibitory and antioxidant activities of SAFE may be involved. Therefore, the observed reduction in SBP and DBP following SAFE administration suggests its potential to attenuate hypertension.

### Hepatotoxicity

**Figure 4:**
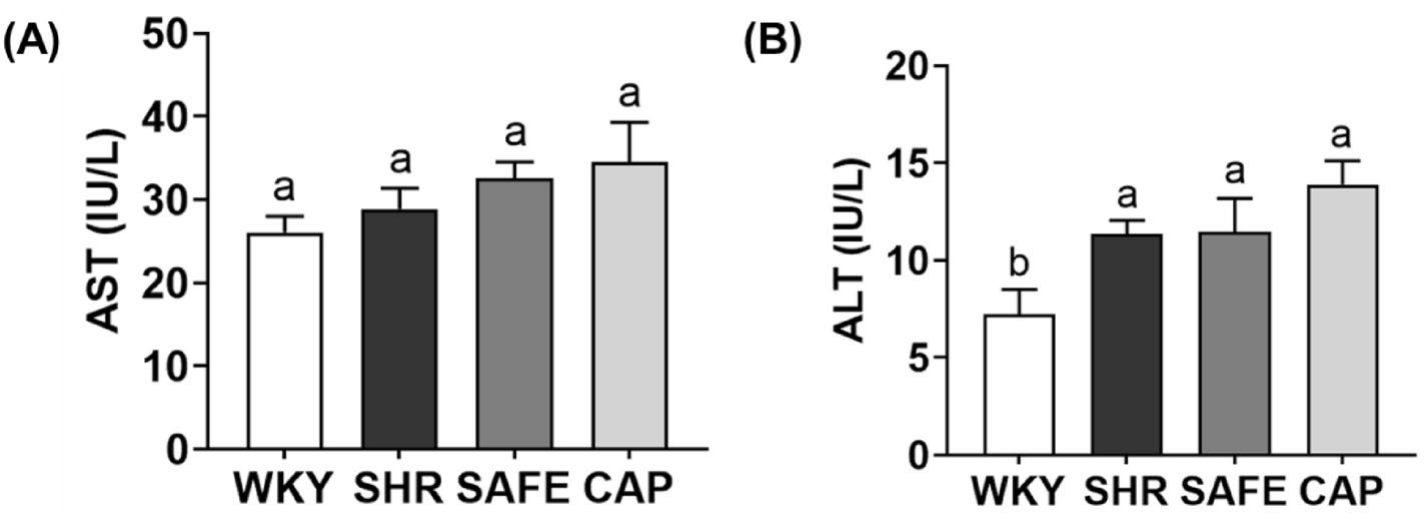
(**A**) Aspartate transaminase and (**B**) alanine aminotransferase levels in the serum of rats supplemented with SAFE. Data are expressed as the mean ± SEM (*n* = 8). Statistical analyses were performed using a one-way ANOVA with the Fisher’s LSD test. Values with different letters indicate significant differences at *p* < 0.05.

As shown in Fig. 4, the serum hepatotoxicity index reveals the AST and ALT levels in all groups ranging from 25.1 to 34.5 and 7.23 to 13.9 IU/L, respectively. These values are within the normal range, indicating the absence of hepatotoxicity^25^. Although the AST levels were not significantly different among the groups, ALT levels were lower in the WKY group than in the other groups. Furthermore, SAFE and CAP exhibited no significant differences in AST and ALT levels compared to that in SHR.

Hypertension can induce liver damage through mechanisms involving inflammation and oxidative stress, resulting in elevated levels of AST and ALT, both of which are released into the bloodstream during liver injury^26^. Previous studies reported higher hepatic enzyme levels in SHR than in WKY^27^. In addition, previous studies demonstrated that each extract showed hepatoprotective effects in animal studies^28,29^. However, in this study, SAFE did not exhibit any hepatoprotective effects. This discrepancy could be attributed to differences in the duration of extract administration or a potential decrease in the number of components with hepatoprotective properties in SAFE. Although SAFE did not demonstrate hepatoprotective effects, we confirmed that SAFE did not induce hepatotoxicity at an administered dose of 500 mg/kg body weight.

### Cardiac mass index and fibrosis

**Figure 5:**
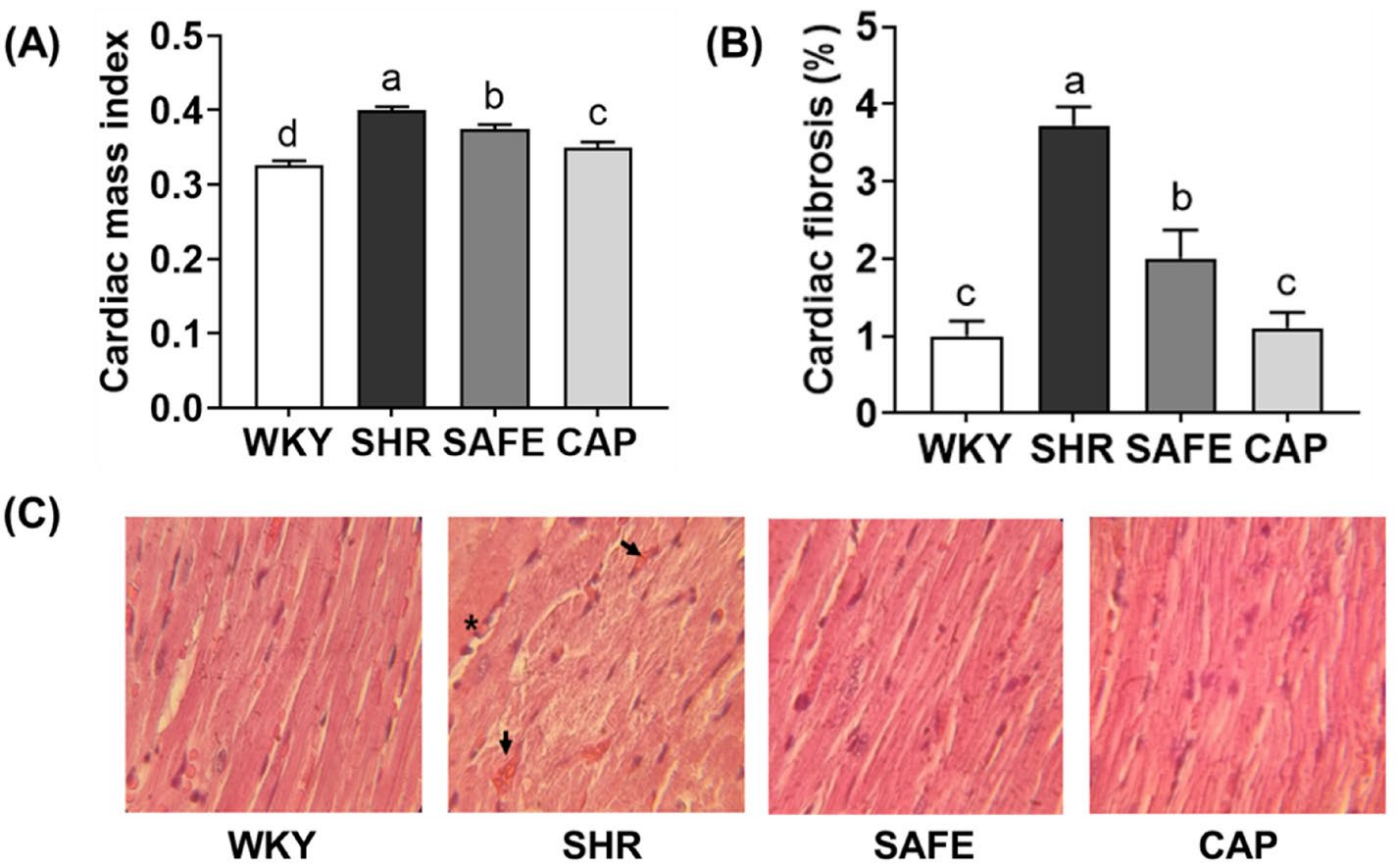
(**A**) Cardiac mass index, (**B**) cardiac fibrosis (%), and (**C**) histology of the cardiac tissues (hematoxylin and eosin stain, ×100) of rats supplemented with SAFE. Each symbol indicates sporadic fragmentation of myocardial fibers (arrows) and eosinophilic staining (stars). Data are expressed as the mean ± SEM (*n* = 8). Statistical analyses were performed using a one-way ANOVA with the Fisher’s LSD test. Values with different letters indicate significant differences at *p* < 0.05.

As shown in Fig. 5A, a significant increase in the cardiac mass index was observed in SHR compared to that in WKY rats (*p* < 0.05). Both SAFE and CAP exhibited significant reductions in cardiac mass index compared to that in SHR, with CAP showing a significantly lower cardiac mass index than that in SAFE (*p* < 0.05). As depicted in Fig. 5B,C, histological analysis of the heart revealed a substantial increase in cardiac fibrosis in SHR compared to that in WKY rats (*p* < 0.05). Moreover, SHR exhibited eosinophil infiltration and fragmentation of myocardial fibers. Significantly, SAFE effectively attenuated cardiac fibrosis compared to that in SHR (*p* < 0.05). Furthermore, CAP demonstrated a remarkable reduction in cardiac fibrosis compared to that in SHR and even in SAFE, comparable to that observed in WKY rats (*p* < 0.05).

Spontaneously hypertensive rats are useful experimental models for studying essential hypertension and heart disease. This model has a distinctive characteristic, notably overactivation of the sympathetic nervous system, which results in organ damage, including cardiac hypertrophy and fibrosis, typically beginning at 6–8 weeks of age^20^. CAP reduces blood pressure by modulating the renin–angiotensin system and protects cardiac cells by inhibiting inflammatory responses and oxidative stress, both associated with heart disease^30,31^. These findings support the significant reduction in cardiac mass index and fibrosis observed in the CAP group in this study. Although no previous studies have investigated the effects of SAFE on cardiac function, their phytochemical components have demonstrated the potential to promote cardiac health. For instance, tannins, which are abundant in sorghum and finger millet, exert antioxidant and anti-inflammatory effects by increasing NO, decreasing endothelin-1, and downregulating angiotensin receptor levels and ERK1/2 phosphorylation, thereby inhibiting cardiac hypertrophy and fibrosis^32,33^. Additionally, vitexin, a compound found in adzuki beans, exerts cardioprotective effects in rats by inhibiting myocardial apoptosis and lipid peroxidation^34^. Catechin, the predominant flavonoid in finger millet, reduces the secretion of tumor necrosis factor and Th2 cytokines, resulting in reduced heart weight and the alleviation of fibrosis^35^. Although the specific compounds in SAFE remain to be identified, the observed reduction in cardiac mass index and improvement in fibrosis may be attributed to the cardioprotective effects of phenolic compounds.

## Conclusions

In conclusion, this study demonstrated that SAFE effectively lowered blood pressure and attenuated cardiac hypertrophy and fibrosis in spontaneously hypertensive rats. However, the chemical composition of SAFE and the mechanisms underlying its antihypertensive effects remain unclear. Therefore, further investigations are necessary to identify the specific active compounds in SAFE and elucidate the mechanisms underlying their antihypertensive effects. Our findings suggest SAFE as a potential dietary supplement for blood pressure regulation and the prevention of cardiovascular disease.

## Data Availability

The datasets used and analyzed during the current study available from the corresponding author on reasonable request.
